# Multiple Gastric Schwannoma: A Case Report

**DOI:** 10.2174/0115734056365968250422110814

**Published:** 2025-05-15

**Authors:** Bin Huang, Mingtai Cao, Xiaoying Zheng, Tuanyue Ma, Yuntai Cao

**Affiliations:** 1 Department of Radiology, Affiliated Hospital of Qinghai University, Xining, China; 2 Department of Pathology, Affiliated Hospital of Qinghai University, Xining, China

**Keywords:** Gastric schwannoma, Multiple imaging features, Magnetic resonance imaging, Computed tomography, Positron emission tomography, Gastrointestinal mesenchymal tumor, Schwann cell

## Abstract

**Background:**

Gastric schwannoma is a rare gastrointestinal mesenchymal tumor with Schwann cell differentiation. In the past, most of the published cases were single gastric schwannoma. Multiple gastric schwannoma is exceedingly rare. We herein report a case of multiple gastric schwannomas.

**Case Presentation:**

A 55-year-old male presented with postprandial vomiting of unclear etiology, accompanied by epigastric pain and bloating. Computed tomography revealed marked thickening of the gastric wall at the fundus-body junction along the greater curvature and gastric angle, with intraluminal nodular projections. Multiphase contrast-enhanced computed tomography demonstrated moderate progressive enhancement. The patient was misdiagnosed as having a gastric stromal tumor before the operation and subsequently underwent laparoscopic partial gastrectomy. However, pathological and immunohistochemical analysis confirmed multiple gastric schwannomas. The patient recovered uneventfully and was discharged without complications.

**Conclusion:**

Gastric schwannoma is rare in clinical practice, especially gastric multiple schwannomas, which are easily confused with gastric stromal tumors, as illustrated in this case, where a preoperative misdiagnosis occurred. Clinicians should enhance their recognition of characteristic imaging features (including Computed tomography, Magnetic resonance imaging, and Positron emission tomography) and employ multimodal diagnostic approaches to optimize preoperative diagnosis.

## 1 INTRODUCTION

Schwannomas are peripheral nerve sheath tumors arising from Schwann cells, histologically classified as benign neuro-genic neoplasms. While schwannomas (neurilemmomas) may occur in any peripheral nerve, they predominantly localize to the head and neck regions, and are rare in the gastrointestinal tract [1]. The most common site of gastrointestinal schwan-noma is the stomach (It accounts for about 0.2% of all gastric tumors.), followed by the rectum and colon, and is rare in the small intestine and esophagus [2]. Gastric schwannoma is mostly single; however, it can be multiple in extremely rare cases. We recently treated a patient with multiple gastric lesions, which were ultimately confirmed to be schwannomas through surgery and pathology. This article focuses on the imaging findings and pathological correlation of this rare lesion, especially in CT imaging, and reviews the relevant literature to discuss differential diagnosis, clinical manage-ment, and prognosis.

## 2 CASE PRESENTATION

A 55-year-old male patient had vomited after eating without obvious inducement before February. The vomitus consisted of digestive juice, white in color, about 5 mL each time, with upper abdominal pain and distending pain. No fever, night sweats, cough, expectoration, chest tightness, or shortness of breath was reported.

The patient underwent gastroscopy at a local hospital on February 1, 2024, suggesting the possibility of gastric stromal tumors. Pathological examination showed inflammatory changes. For further treatment, the patient visited the outpatient department of our hospital on February 19, 2024. The patient had a healthy past, no history of chronic diseases, and physical examination was negative.

**Fig. (1):**
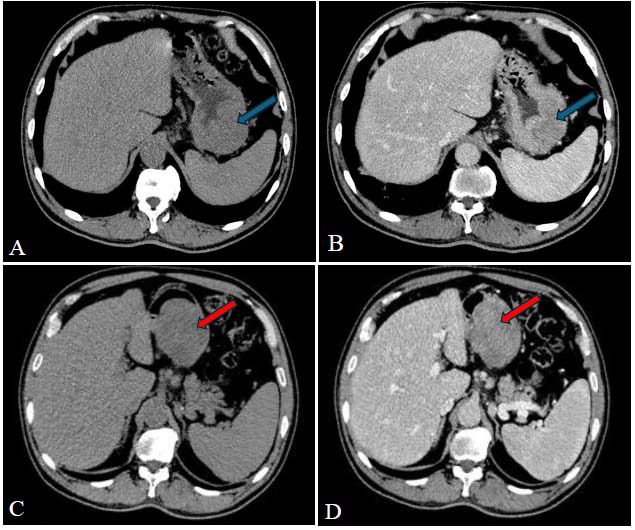
CT cross-section of the lesion. The blue arrows in (**A**) and (**B**) show the CT plain scan and enhanced images of gastric body-gastric fundus lesions, respectively. The density of the plain scan lesions is slightly lower than the muscle density, and the enhanced scan is moderately enhanced. The red arrows in (**C**) and (**D**) show the CT plain scan and enhanced images of the gastric angle lesions, respectively. The enhancement shows moderate enhancement, the low-density area is gradually strengthened, and the enhancement gradually becomes uniform.

**Fig. (2):**
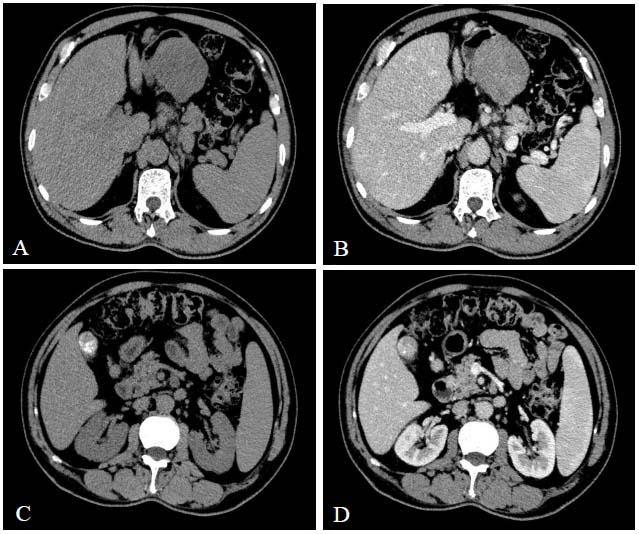
CT cross section of enlarged lymph nodes. The red arrows in (**A**) and (**B**) show enlarged lymph nodes near the gastric angle, and the enhanced scan shows obvious enhancement. The blue arrows and purple arrows in (**C**) and (**D**) show enlarged lymph nodes near the hepatogastric space and retroperitoneal large blood vessels, respectively, and the enhanced scan shows obvious enhancement.

Laboratory tests revealed C-reactive protein (CRP) at 97.39 mg/L and procalcitonin at 4.63 ng/mL (increased). After admission, an abdominal CT examination showed that the gastric wall at the greater curvature side of the gastric fundus-body junction and the gastric angle were significantly thickened, with nodular protrusions extending into the lumen (Fig. **1**). The sizes were approximately 76 mm × 39 mm × 51 mm and 72 mm × 58 mm × 46 mm. The plain scan revealed uniform density, with small patchy areas of slight heterogeneity (Fig. **2**). The three-phase enhanced scan showed moderate progressive enhancement. Multiple enlarged lymph nodes were seen near the gastric angle, the hepatogastric space, and the retroperitoneal great vessels. The short diameter of the largest lymph node was 12 mm, and the enhanced scan showed significant enhancement. The preoperative diagnosis was a gastric stromal tumor. After excluding surgical contrain-dications, laparoscopic partial gastrectomy was performed under general anesthesia in our hospital on February 26, 2024.

**Fig. (3):**
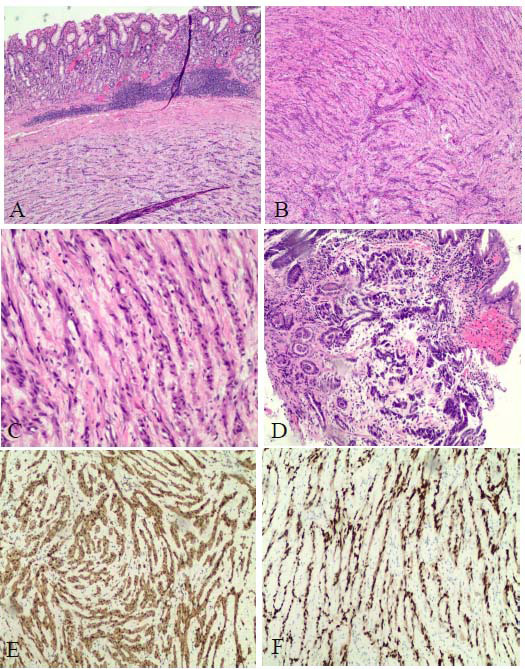
Pathologic and immunohistochemical results of the patient. (**A**): The tumor is located in the submucosa of the gastrointestinal tract, with a clear boundary with the gastrointestinal tract, and a large number of lymphocytes infiltrated around the tumor; (**B**): The tumor is composed of Antoni A and Antoni B regions; (**C**): Spindle Schwann cells show cord-like hyperplasia. Collagen fibers are seen between the cells. Some tumor cells are mildly atypical, but no mitosis is observed. (**D**): Gland structure is rare. Most of the cancer cells are scattered, nest-like growths. Cell heteromorphism is obvious; nuclear size is different, deep staining, nucleolus is obvious, nuclear division can be seen; (**E**): The tumor cells show diffuse strong positive expression of S100; (**F**): The tumor cells are diffusely strongly positive for SOX10.

The postoperative pathological examination confirmed a spindle cell tumor of gastric origin (Fig. **3**). Integrated with immunohistochemical findings, the diagnosis was consistent with schwannoma. Immunohistochemical profiles demons-trated the following: CK(-), CD34(-), CD117(-), DOG-1(-), SDHB (retained), PHH3(-), S100(+), SOX-10(+), CD56(focal+), SMA(-), Des(-), and Ki67(<10%). The postoperative course was uneventful, with a marked response to supportive care. The patient achieved a stable clinical condition and was able to self-discharge after several days. Regular follow-up was advised.

## 3 DISCUSSION

Schwannoma arises from the uncontrolled proliferation of nerve sheath Schwann cells. While these tumors may arise in any anatomical region, gastrointestinal involvement is uncommon [3]. Gastrointestinal schwannoma (GS) was first described by Daimaru *et al*. in 1988 [4]. The most common site of gastrointestinal schwannoma was the stomach, followed by the colon and rectum. Gastric schwannoma is mainly derived from the Schwann cells of the gastric submucosal plexus-Meissner plexus and the myenteric plexus-Auerbash plexus [5]. It is often benign and grows slowly, accounting for about 0.2% of all gastric tumors. In recent years, more and more gastric schwannomas have been found and reported, but almost all cases are single and multiple cases are rare. In this case, only multiple schwannomas were found in the stomach, with no associated abnormalities in the skin or hearing. Additionally, no molecular analysis was performed. Therefore, a preliminary diagnosis of “schwannomatosis, not otherwise specified (NOS)” can be considered [6]. Given the paucity of literature on multifocal GS, the following review focuses on the general characteristics of GS.

Gastric schwannoma is more common in women aged 50-60 years old [7]. Tumor progression is typically accompanied by nonspecific clinical manifestations.

Over 98% of cases are benign [1]. Most of them are found by chance during routine physical examination. Only a few patients may have upper abdominal discomfort, abdominal pain, gastrointestinal bleeding, and other symptoms. In select cases, the patient may experience vomiting due to obstruction caused by a larger tumor in the gastric corner.

Pathology and immunohistochemistry currently serve as the gold standard for diagnosing Gastric Schwannoma (GS). The histopathological features of GS exhibit characteristic patterns, *i.e*., tumors typically arise in the submucosa, presen-ting as well-circumscribed solid masses with homogeneous cut surfaces. Secondary degenerative changes, such as cystic trans-formation, necrosis, or hemorrhage, are rarely observed [8].

Microscopically, spindle cells are arranged in trabecular and microfascicular patterns, lacking the nuclear palisading and Verocay bodies typically seen in soft tissue schwannomas. Peritumoral lymphocytic infiltration with lymphoid follicle formation represents a hallmark diagnostic feature [9].

Immunohistochemically, GS demonstrates consistent S100 and SOX10 positivity, while CD34 typically shows negativity or focal reactivity. DOG-1 remains uniformly negative [8, 10]. This profile contrasts with Gastrointestinal Stromal Tumors (GISTs) and leiomyomas. GISTs exhibit CD34, CD117, and DOG-1 positivity (DOG-1 being the most sensitive marker) [11], whereas leiomyomas demonstrate SMA and desmin expression [12].

The characteristics of GS in imaging findings show that the tumor is more common gastric body, followed by antrum and gastric fundus. Growth patterns may include intraluminal, extraluminal, or mixed configurations. CT typically demonstrates a round or oval submucosal mass protruding from the gastric wall, with well-defined margins and intact overlying mucosa.

The internal density is relatively homogeneous, often slightly hypodense compared to muscle, and rarely shows liquefactive necrosis or cystic degeneration. Enhancement patterns reveal mild-to-moderate progressive homogeneous enhancement [13]. As malignant GS cases are exceedingly rare, it remains unclear whether malignant variants are more prone to cystic changes, warranting further investigation. In some cases, enlarged perigastric and retroperitoneal lymph nodes exhibiting avid enhancement may represent reactive hyperplasia rather than malignant metastasis [14-16]. MRI findings of GS are sparsely reported. Compared to CT, MRI better delineates tumor boundaries. On MRI, GS appears isointense to hypointense on T1-weighted imaging (T1WI) and hyperintense on T2-weighted imaging (T2WI). T2WI clearly demarcates the tumor from the mucosal layer, aiding in identifying its submucosal origin. Contrast-enhanced MRI demonstrates progressive enhancement. Restricted diffusion (high signal on DWI with reduced ADC values) may suggest malignant potential [17]. GS exhibits a high uptake of [^18^F]-FDG on PET imaging [17], which may be related to the positive expression of autocrine motility factors in immunohistochemistry [18].

Given the distinct imaging characteristics of GS, CT with multiplanar reconstruction (MPR) provides clear visualization of the tumor's spatial relationships with adjacent structures. Compared to MRI and PET, CT offers superior cost-effectiveness. The following section briefly discusses the key points for identifying GS on CT:

(1) **GIST:** The tumor is often spherical, round-like, and may be irregular when it is large. The tumor is prone to cystic necrosis, hemorrhage, and uneven density. After enhancement, the tumor is often moderately enhanced. When combined with necrosis and cystic change, it shows uneven enhancement; compared with GS, GIST has less peritumoral lymph node enlargement, which can be used as a key point in the identification of GS. In addition, related studies have shown that the edge of GIST lesions is more blurred than that of GS, and the tumor doubling time is shorter. GS mostly shows mixed growth inside and outside the gastric wall, while GIST rarely shows this growth pattern [19, 20]. However, distinguishing gastric GS from small benign and malignant GIST without secondary changes remains challenging. This requires correlation with postoperative pathology and immunohistochemistry for accurate diagnosis.

(2) **Leiomyoma:** It occurs in the cardia and involves the gastroesophageal junction. It has mainly intracavitary growth and has the characteristics of growing along the gastric wall. The surface of the mass is smooth or slightly lobulated, and there is a clear boundary with the normal gastric wall. The density of the CT plain scan is uniform, and the enhanced scan shows mild to moderate uniform enhancement.

(3) **Gastric lymphoma:**The main imaging manifestations of gastric lymphoma are gastric wall thickening and the presence of a mass, while gastric stenosis is less common. Retroperitoneal lymph node enlargement at the level of renal hilum without perigastric lymph node enlargement is the characteristic manifestation of primary gastric malignant lymphoma.

(4) **Gastric cancer:** GS with larger or secondary changes, such as ulcers, should be distinguished from gastric cancer. Gastric cancer often involves the adjacent gastric wall, resulting in thickening and stiffness with enlarged lymph nodes around the tumor and becoming prone to distant metastasis. In contrast, GS occurs in the submucosa, with a limited scope and a clear boundary with the gastric wall.

Currently, it remains difficult to detect schwannomas directly through imaging alone. Endoscopy can identify tumors involving the gastric mucosa, such as gastric cancer; however, tumors located in other parts, such as the muscular layer (muscularis propria) are difficult to observe with conventional endoscopy. In these cases, endoscopic ultrasonography (EUS) offers significant advantages, allowing for precise evaluation of submucosal lesions. Additionally, studies have shown that Endoscopic Ultrasound-guided Fine Needle Aspiration (EUS-FNA) can effectively differentiate gastric schwannomas from gastrointestinal stromal tumors [21].

However, due to technical limitations at our institution, this procedure was not implemented, resulting in a lack of a definitive preoperative diagnosis. Many hospitals face similar challenges, underscoring the need to adopt more advanced diagnostic techniques for difficult-to-identify gastric submucosal tumors. Comprehensive diagnostic approaches (including imaging, endoscopy, etc) may improve preoperative diagnostic accuracy, thereby facilitating the development of timely, individualized treatment plans for patients.

## CONCLUSION

In summary, Gastric Schwannoma (GS) remains a rare clinical entity with a high preoperative misdiagnosis, frequently confused with gastrointestinal stromal tumors (GISTs). GS typically demonstrates favorable long-term outcomes, whereas 10–30% of GISTs exhibit malignant behaviors, such as recurrence. These distinct biological profiles necessitate differential therapeutic strategies and prognostic assessments. Accurate preoperative diagnosis may prevent overtreatment. Although genetic testing was not performed in this case, the clinical presentation aligns with “schwannoma-tosis-not otherwise specified (NOS).” Enhanced recognition of this disease among radiologists and clinicians through multidisciplinary collaboration could reduce diagnostic errors and improve diagnostic accuracy.

## AUTHORS' CONTRIBUTIONS

H.B.: Conceived the study; C.M.T., Z.X.Y., and M.T.Y.: Helped to collect relevant information; C.Y.T.: Helped to review and modify the paper.

## Data Availability

The data and supportive information are available within the article.

## LIST OF ABBREVIATIONS

CT: Computed Tomography
MRI: Magnetic Resonance Imaging
GIST: Gastrointestinal Stromal Tumor
EUS: endoscopic ultrasonography
GS: Gastrointestinal schwannoma
MPR: Multiplanar reconstruction

## STANDARDS OF REPORTING

CARE guidelines and methodologies were followed.
